# Author Correction: Efficient and stable visible-light-driven Z-scheme overall water splitting using an oxysulfide H_2_ evolution photocatalyst

**DOI:** 10.1038/s41467-024-45596-2

**Published:** 2024-02-07

**Authors:** Lihua Lin, Yiwen Ma, Junie Jhon M. Vequizo, Mamiko Nakabayashi, Chen Gu, Xiaoping Tao, Hiroaki Yoshida, Yuriy Pihosh, Yuta Nishina, Akira Yamakata, Naoya Shibata, Takashi Hisatomi, Tsuyoshi Takata, Kazunari Domen

**Affiliations:** 1 Research Initiative for Supra-Materials, Interdisciplinary Cluster for Cutting Edge Research, Shinshu University, Nagano, Japan; 2 Institute for Engineering Innovation, The University of Tokyo, Bunkyo-ku, Tokyo Japan; 3 Science and Innovation Center, Mitsubishi Chemical Corporation, Aoba-ku, Yokohama-shi, Kanagawa Japan; 4 Japan Technological Research Association of Artificial Photosynthetic Chemical Process (ARPChem), Tokyo, Japan; 5 Office of University Professors, The University of Tokyo, Bunkyo-ku, Tokyo Japan; 6 Graduate School of Natural Science and Technology, Okayama University, Kita-ku, Okayama Japan; 7 Faculty of Natural Science and Technology, Okayama University, Kita-ku, Okayama Japan

**Keywords:** Photocatalysis, Energy

Correction to: *Nature Communications* 10.1038/s41467-024-44706-4, published online 09 January 2024

The original version of this Article contained an error in the Methods section, within the “Preparation of BVO powder by hydrothermal method” subsection and incorrectly read “A precursor solution was prepared by dissolving 10.0 mmol of NH_4_VO_3_ (1.170 g, Wako) and 10.0 mmol of Bi(NO_3_)_3_·5H_2_O (4.851 g, Wako) in a 2.0 mM nitric acid solution (60 mL, Wako), the pH of which was adjusted to approximately 0.5 by adding an ammonia solution (28.0–30.0 wt%, Wako).”. The correct version states “2.0 M” instead of “2.0 mM”. This does not change the results or conclusions reported in the manuscript.

These errors have been corrected in both the PDF and the HTML versions of the Article.

