# Supplementary material for: Efficient and stable visible-light-driven Z-scheme overall water splitting using an oxysulfide H2 evolution photocatalyst
Source: Nat Commun. 2024 Jan 9;15:397. doi: 10.1038/s41467-024-44706-4 (PMC10776739; doi:10.1038/s41467-024-44706-4)
Supplement: Supplementary file 1 — Supplementary Information [file 41467_2024_44706_MOESM1_ESM.pdf]

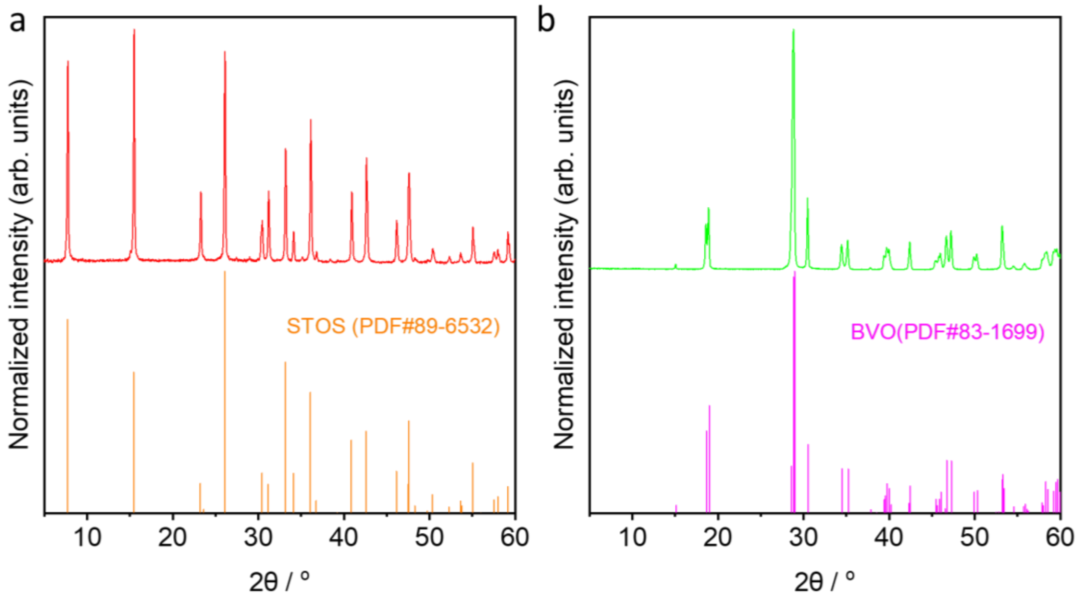

Supplementary Figure 1. Characterization of the HEP and OEP. XRD patterns obtained from the **a,** STOS and **b,** BVO.

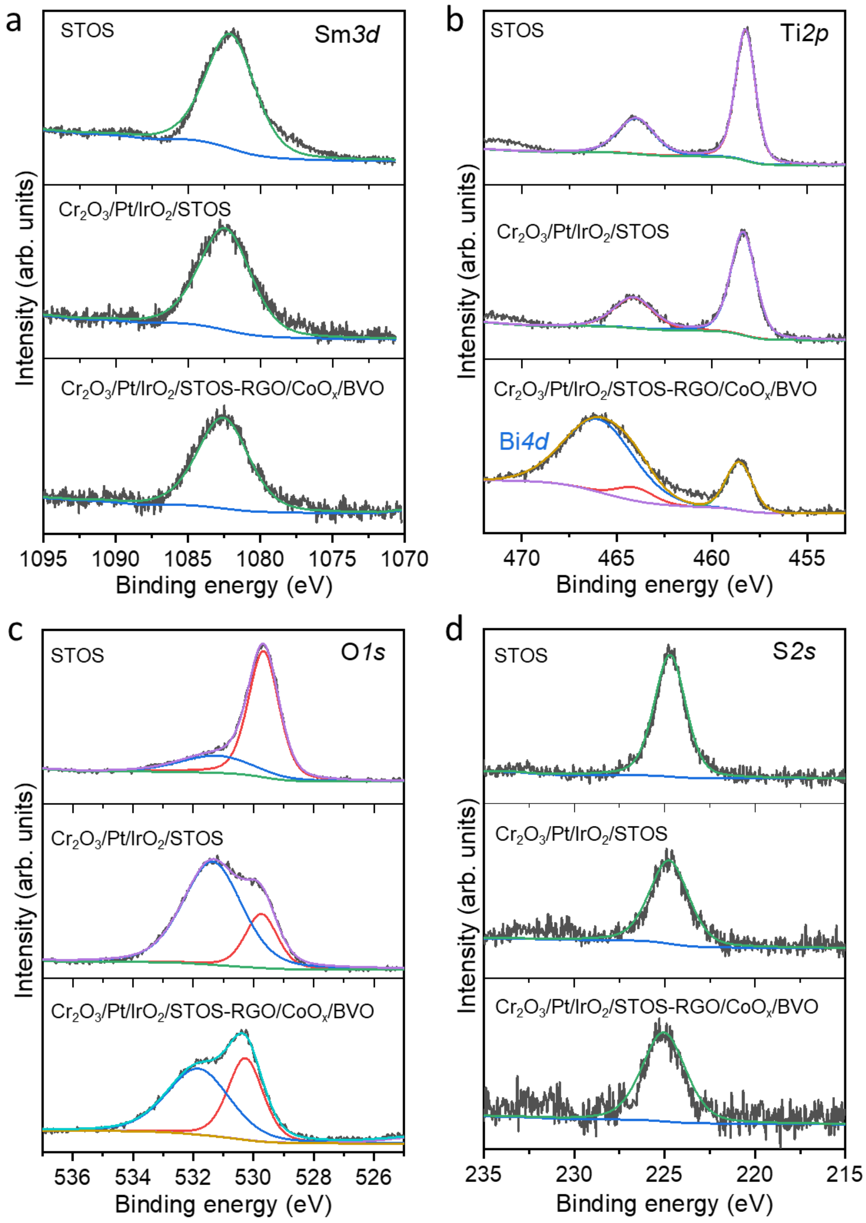

Supplementary Figure 2. Analysis of chemical states of elements in the HEP. The XPS patterns of **a,** Sm3*d*, **b,** Ti2*p*, **c,** O1*s* and **d,** S2*s* XPS data acquired from STOS, Cr_2O_3/Pt/IrO_2/STOS and Cr_2O_3/Pt/IrO_2/STOS-RGO/CoO_x/BVO.

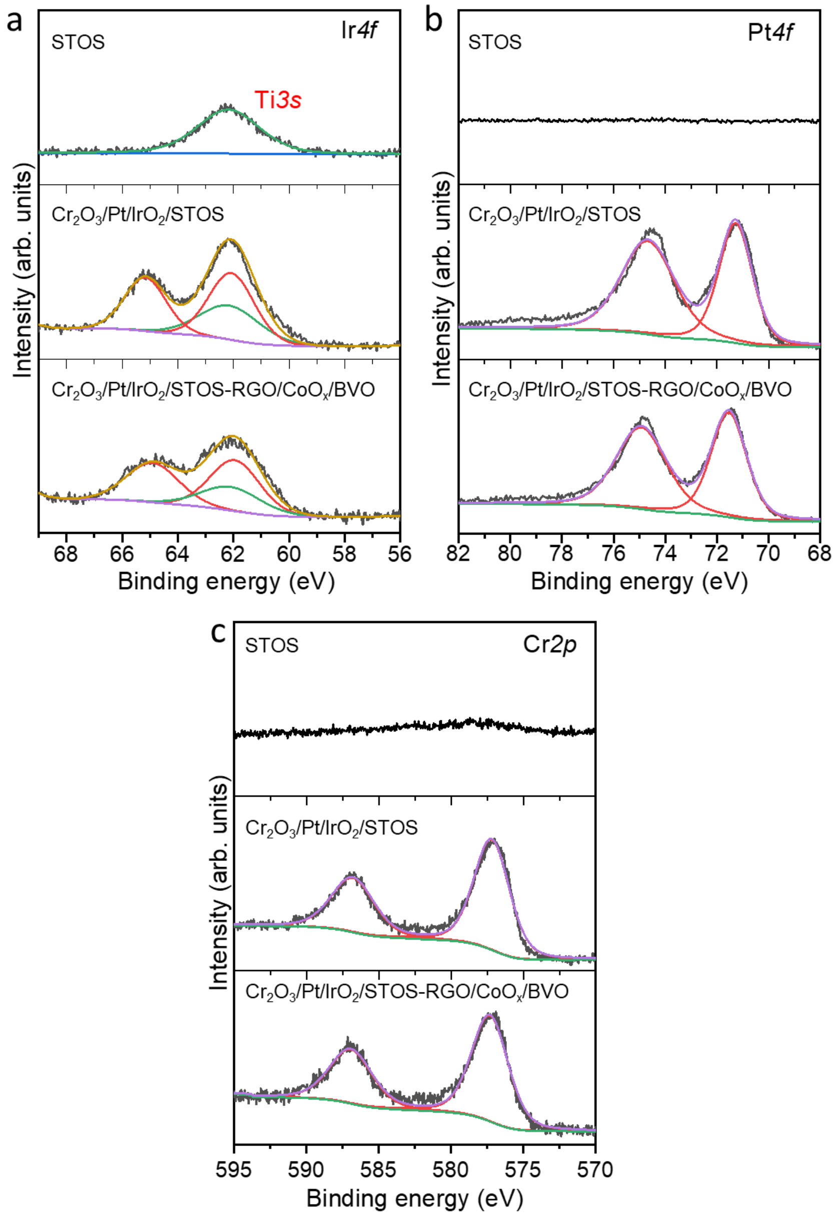

Supplementary Figure 3. Analysis of chemical states of elements in HEP. The XPS patterns of **a**, Ir4*f*, **b**, Pt4*f* and **c**, Cr2*p* XPS data acquired from STOS, Cr_2O_3/Pt/IrO_2/STOS and Cr_2O_3/Pt/IrO_2/STOS-RGO/CoO_x/BVO.

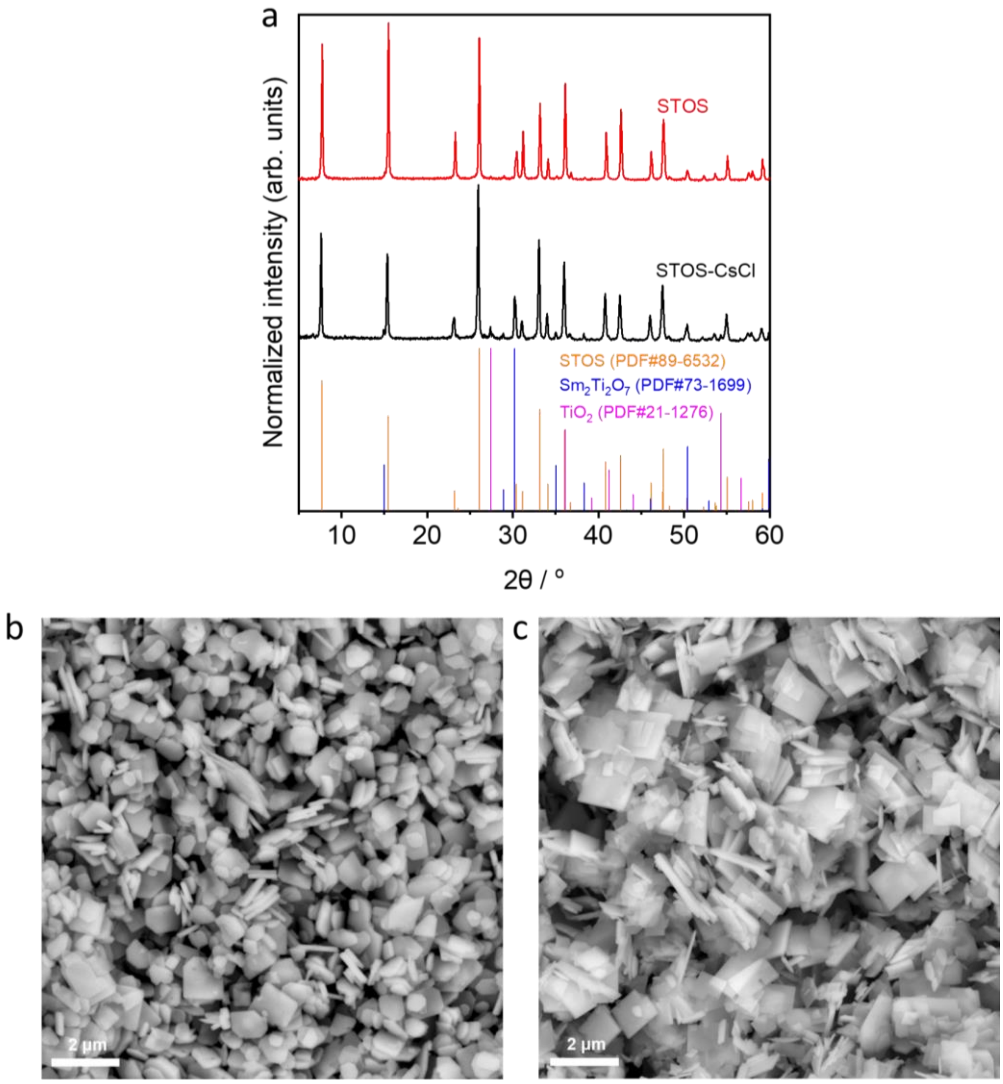

Supplementary Figure 4. Characterization of STOS prepared using a CsCl flux. a, XRD pattern for sample. SEM images of STOS prepared using **b,** $CaCl_2$/LiCl and **c,** CsCl fluxes.

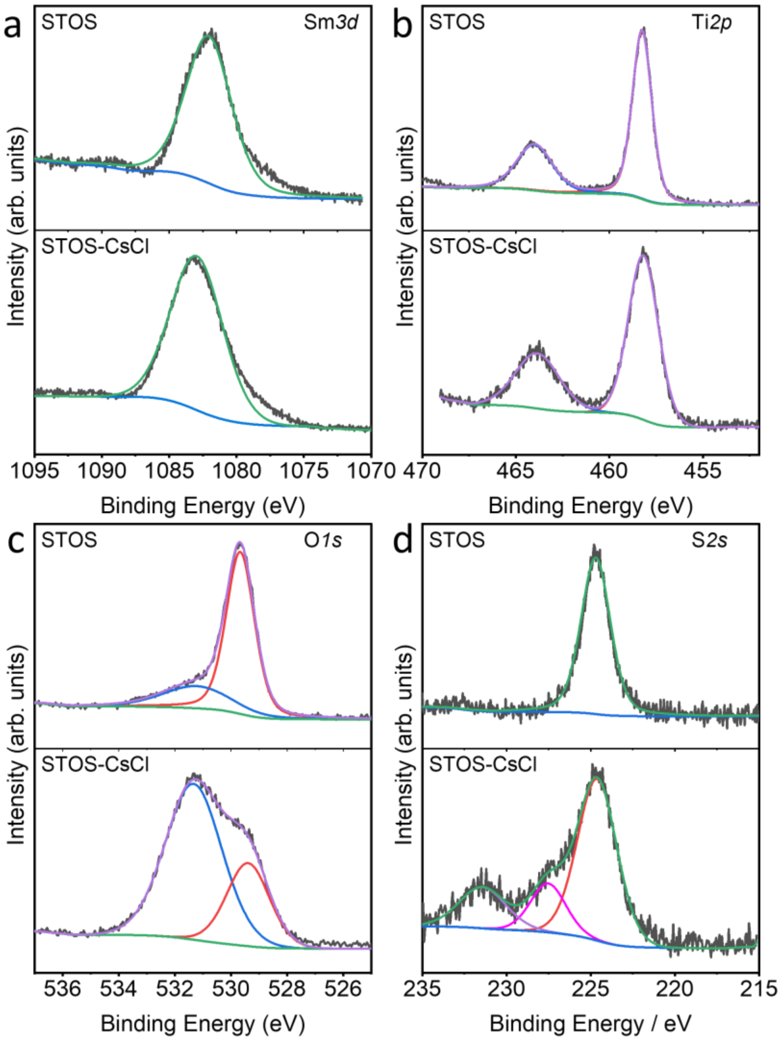

Supplementary Figure 5. Analysis of chemical states of elements in STOS-CsCl. The XPS patterns of **a**, Sm3*d*, **b**, Ti2*p*, **c**, O1*s*, **d**, S2*s* with the XPS signals of STOS as the reference.

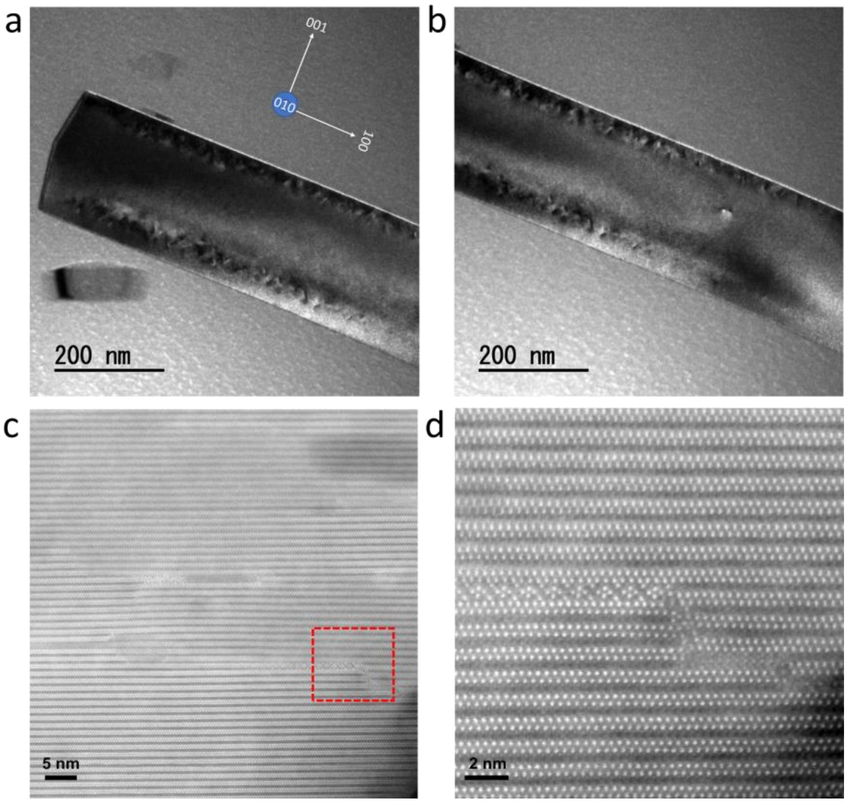

Supplementary Figure 6. Analysis of STOS-CsCl crystal. a, b, Dark field TEM images of STOS-CsCl. **c,** Atomic resolution ADF images STOS-CsCl and **d,** the corresponding enlarged area.

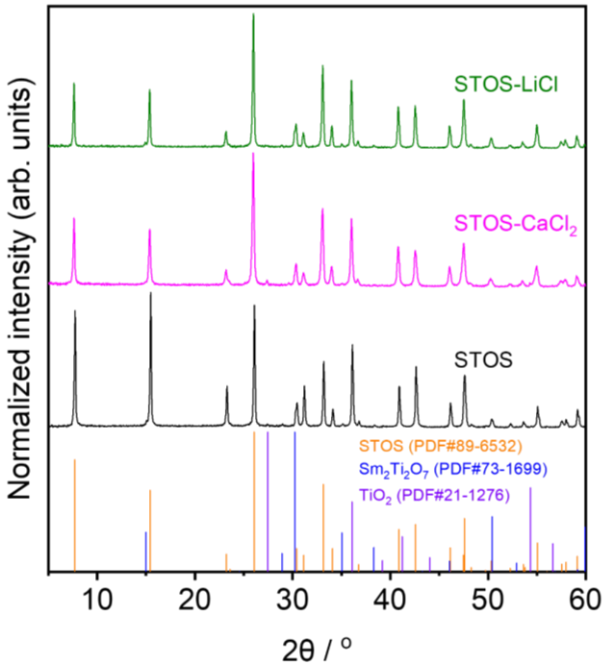

Supplementary Figure 7. Analysis of STOS-CsCl crystal. XRD patterns of STOS-$CaCl_2$ and STOS-LiCl with the peak position of STOS, $Sm_2Ti_2O_7$ and TiO_2 as references.

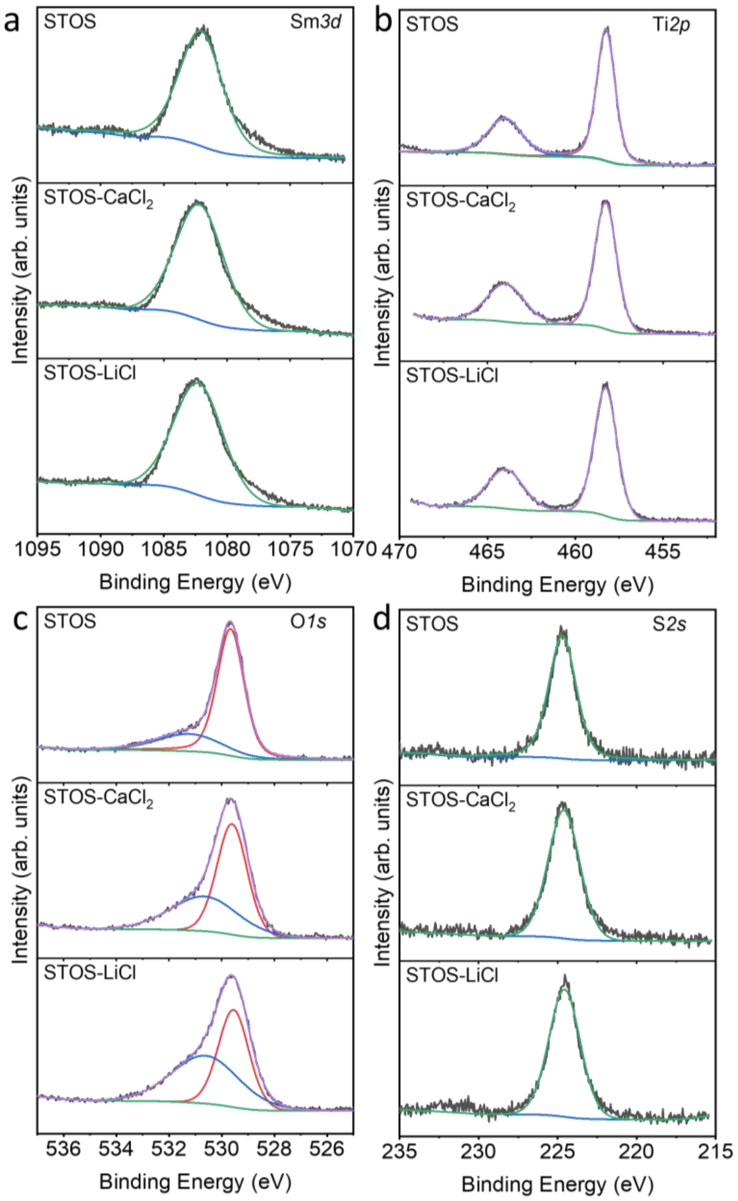

Supplementary Figure 8. Analysis of chemical states of elements in STOS-CaCl₂ and STOS-LiCl. The XPS patterns of **a**, Sm3*d*, **b**, Ti2*p*, **c**, O1*s*, **d**, S2*s* with the signals of STOS as the reference.

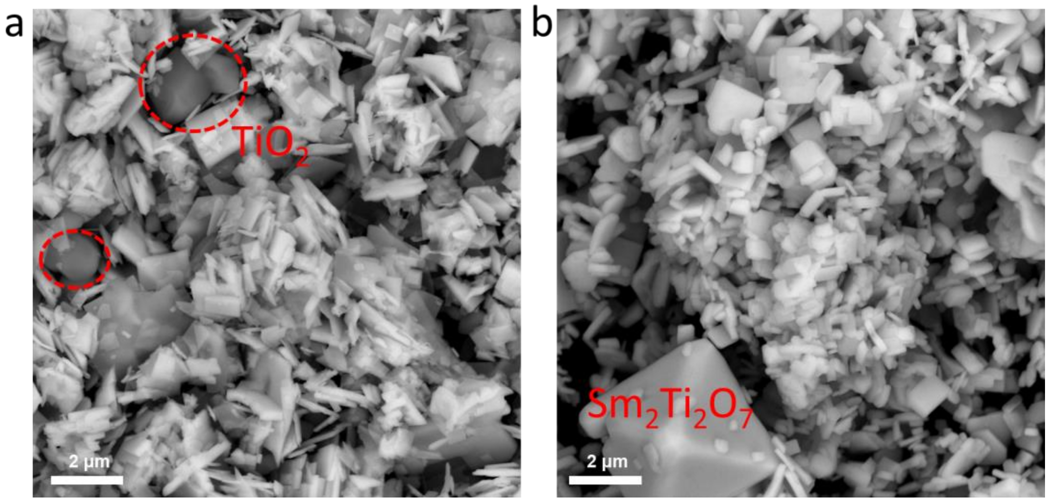

Supplementary Figure 9. Analysis of the morphology of STOS prepared by different flux. SEM images of **a**, STOS-$CaCl_2$, **b,** STOS-LiCl.

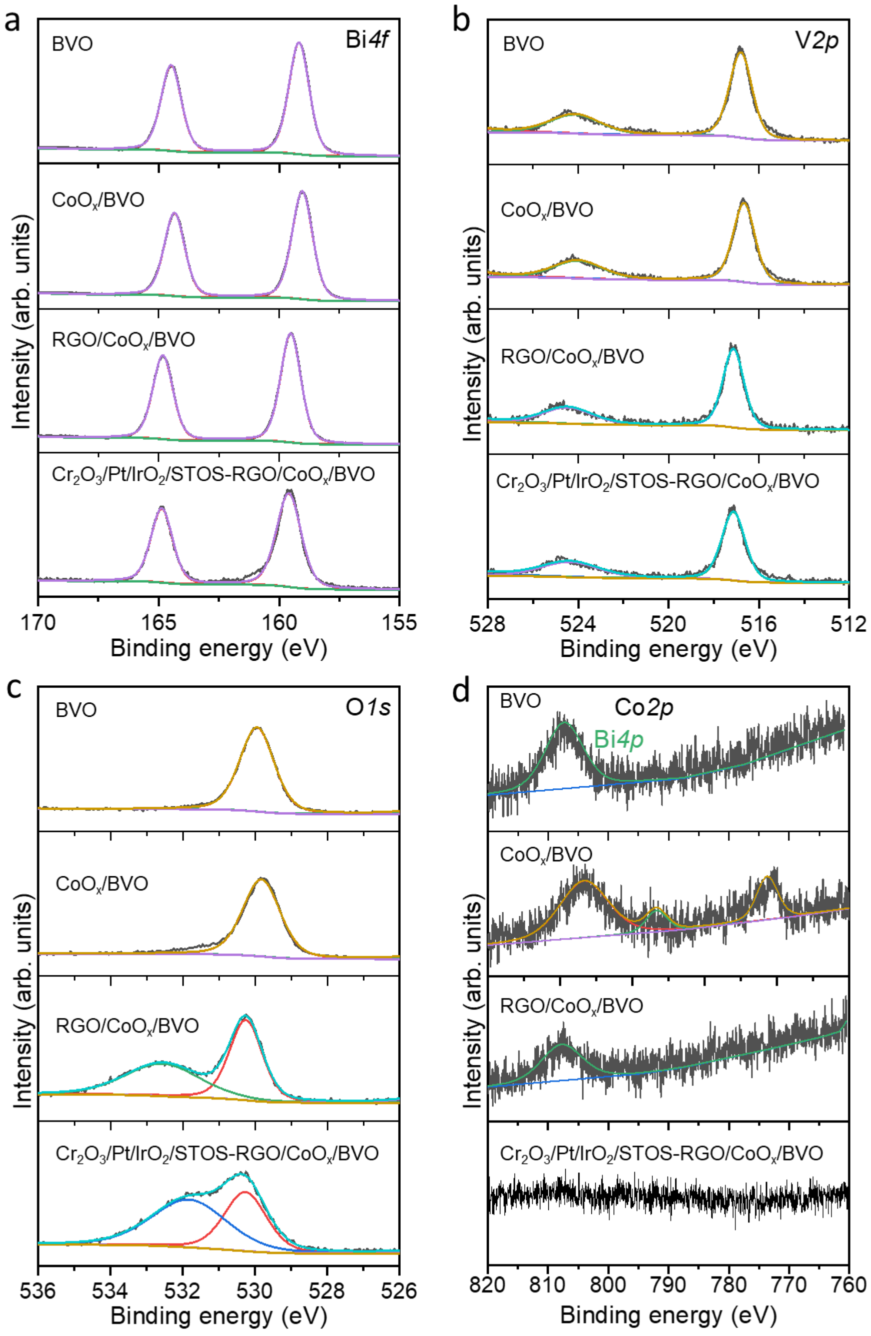

Supplementary Figure 10. Chemical state of elements in OEP. The XPS patterns of **a**, Bi4*f*, **b**, V2*p*, **c**, O1*s* and **d,** Co2*p* XPS data acquired from BVO, CoO_x/BVO and Cr_2O_3/Pt/IrO_2/STOS-RGO/CoO_x/BVO.

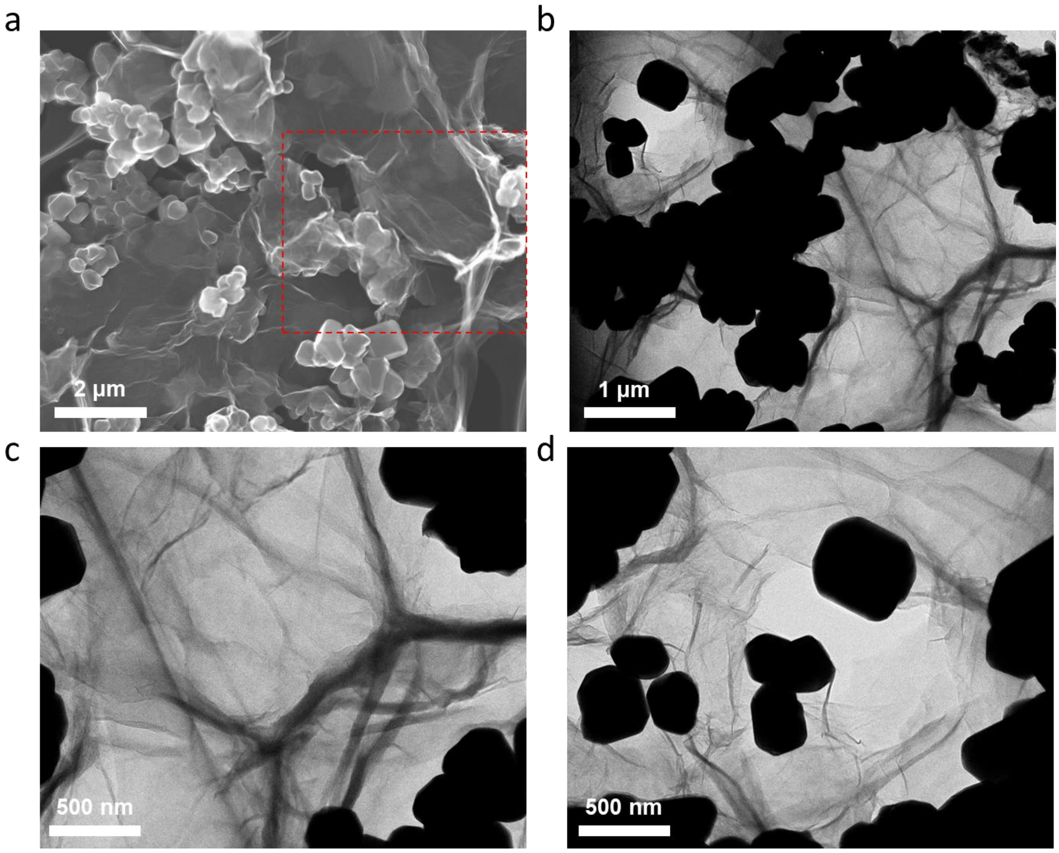

Supplementary Figure 11. Morphology of RGO. a, An SEM image of an $RGO/CoO_x/BVO$ specimen and **b-d,** the corresponding TEM images.

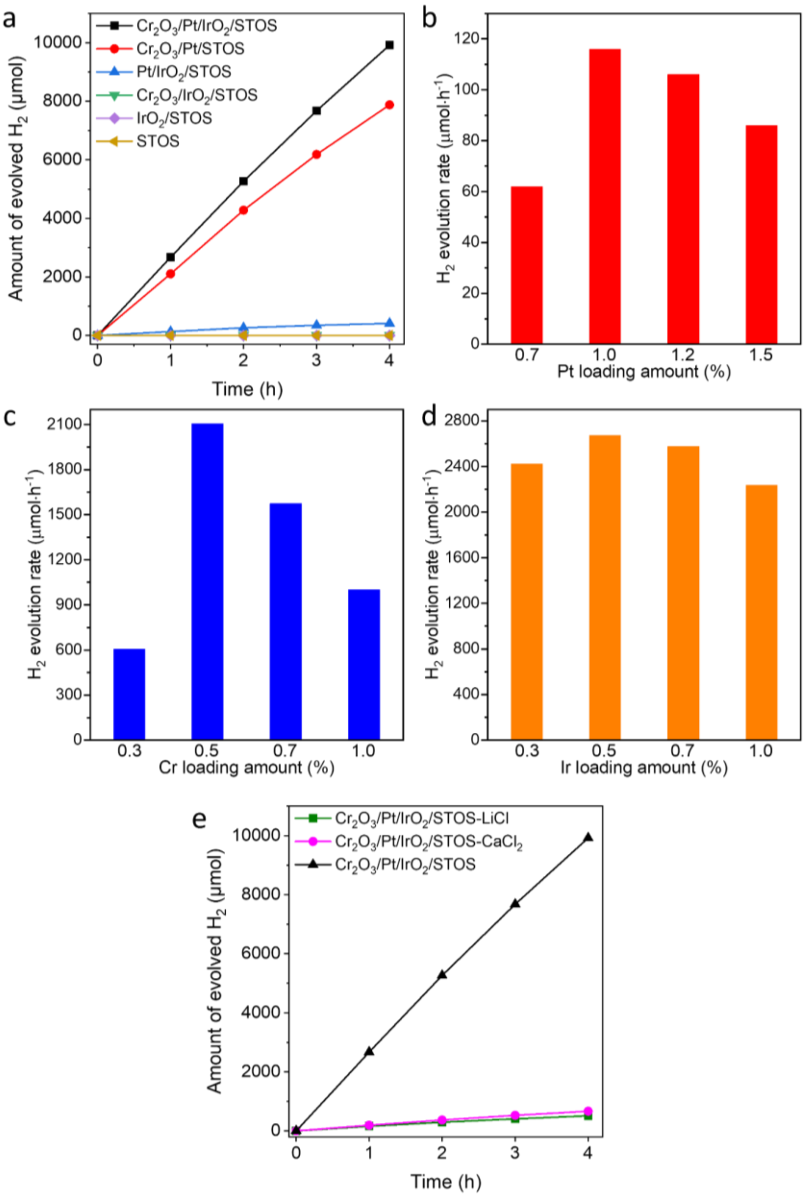

Supplementary Figure 12. H_2 evolution performance obtained from STOS. a, Amounts of H_2 evolved over bare STOS and STOS specimens with different surface modifications over time. **b,** H_2 evolution rate of STOS loading with different amount of Pt. **c,** H_2 evolution rate of 1.0 wt% Pt modified STOS with different loading amount of Cr. **d,** H_2 evolution rate of 1.0 wt% Pt and 0.5 wt% Cr modified STOS with different loading amount of Ir, **e,** Amounts of H_2 evolved over $Cr_2O_3/Pt/IrO_2$ modified STOS, STOS-$CaCl_2$ and STOS-LiCl.

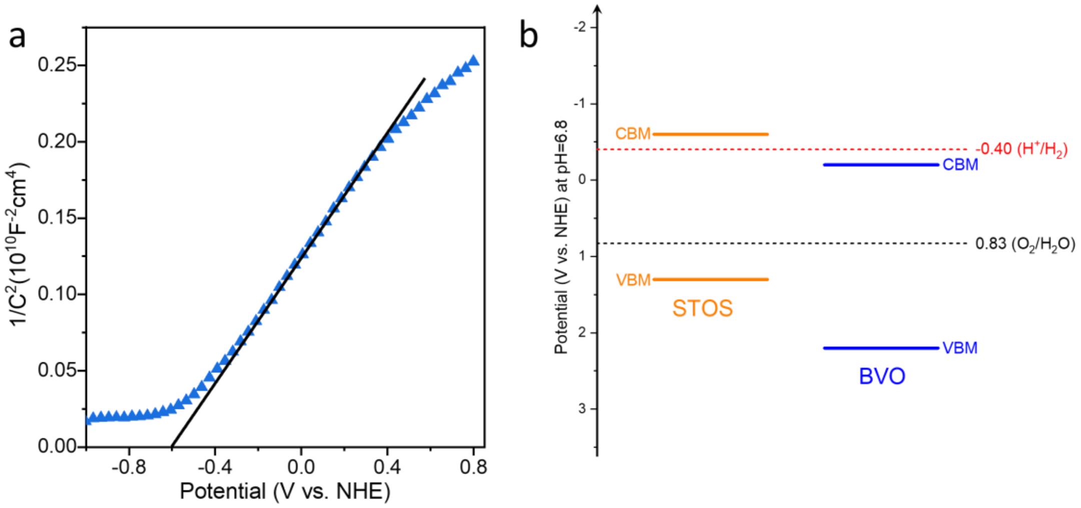

Supplementary Figure 13. Band position of the Z-scheme system. a, Mott-Schottky plot of STOS. **b,** Band alignment of the STOS and BVO.

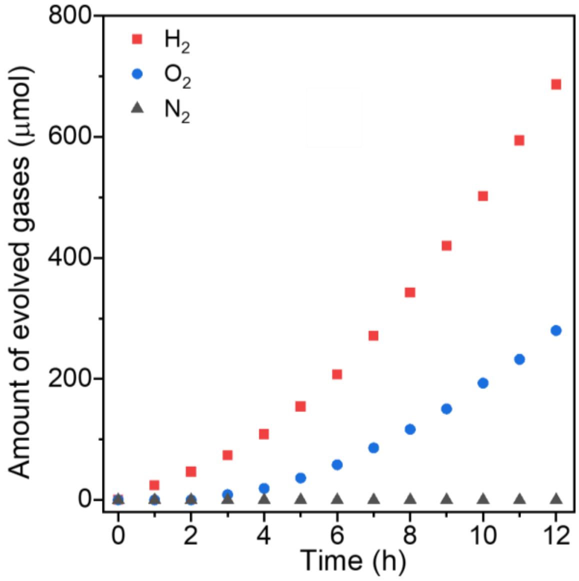

Supplementary Figure 14. Progress of OWS reaction. The evolution of various gases from a Z-scheme system during the induction period as functions of time.

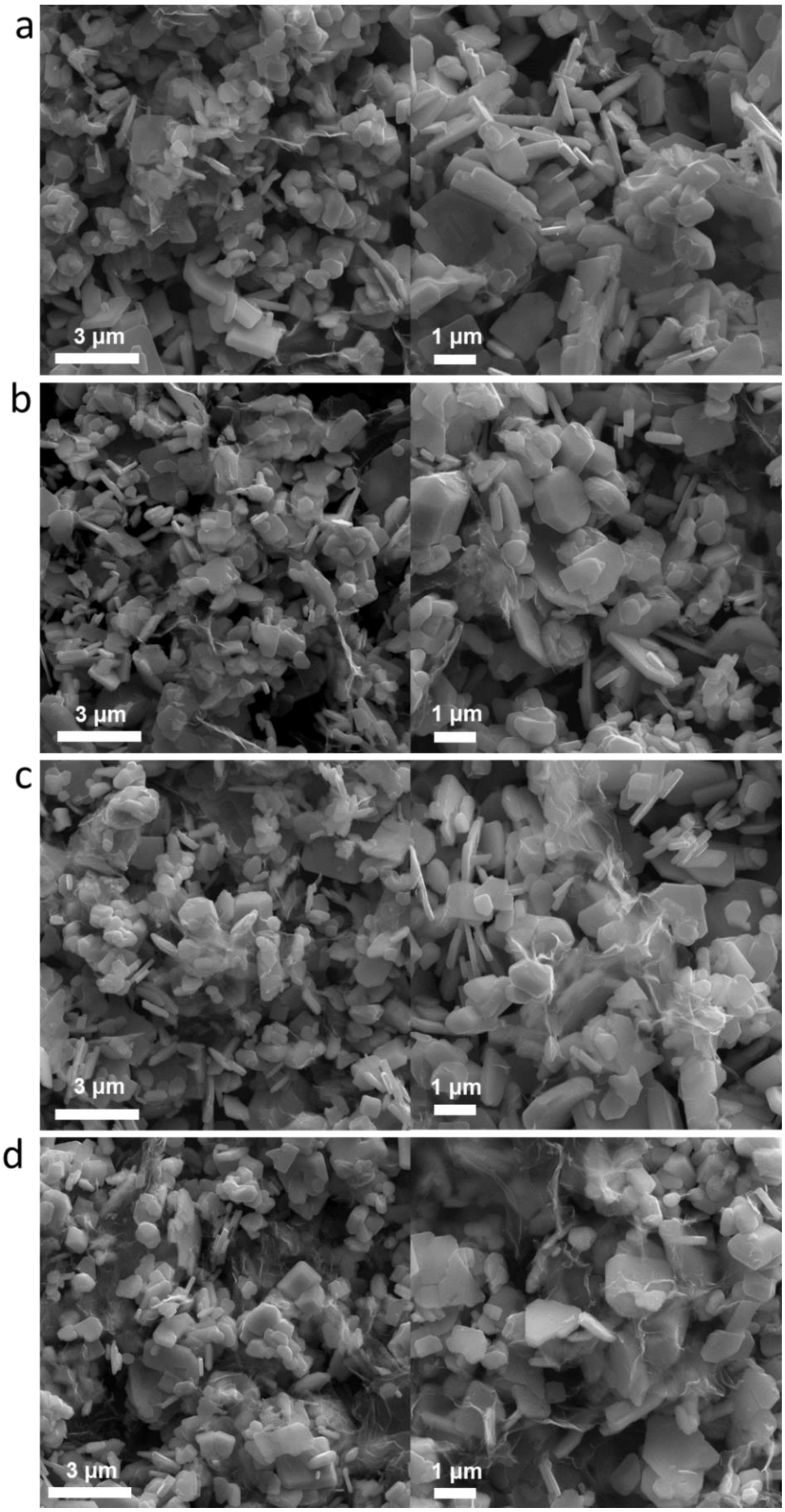

Supplementary Figure 15. Morphology of catalyst specimen during induction period. SEM images of the Cr_2O_3/Pt/IrO_2/STOS-RGO/CoO_x/BVO after exposure to visible light (>420 nm) for **a**, 0, **b**, 4, **c**, 8 and **d**, 12 h.

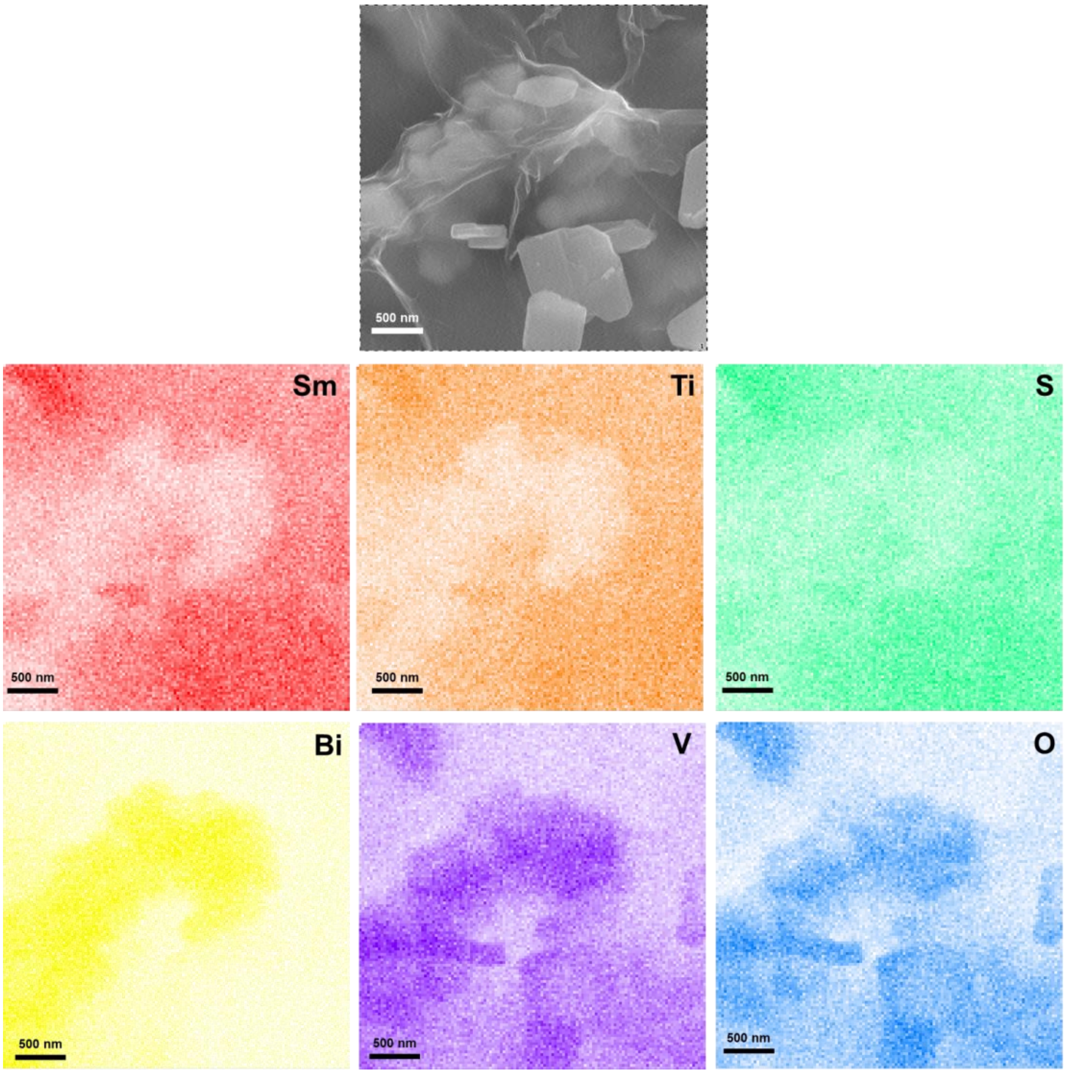

Supplementary Figure 16. Elemental distribution in Z-scheme system. EDS mapping of the elements in the Z-scheme system and the stack of the distribution of Sm and Bi.

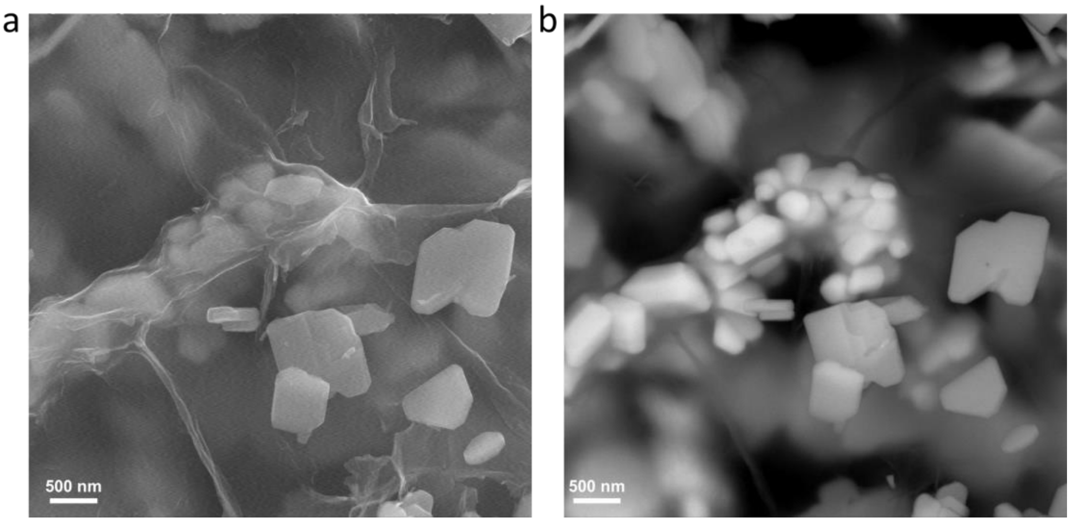

Supplementary Figure 17. Morphology of the Z-scheme system. SEM of Z-scheme system by **a,** secondary electron detector and **b,** back scattering detector.

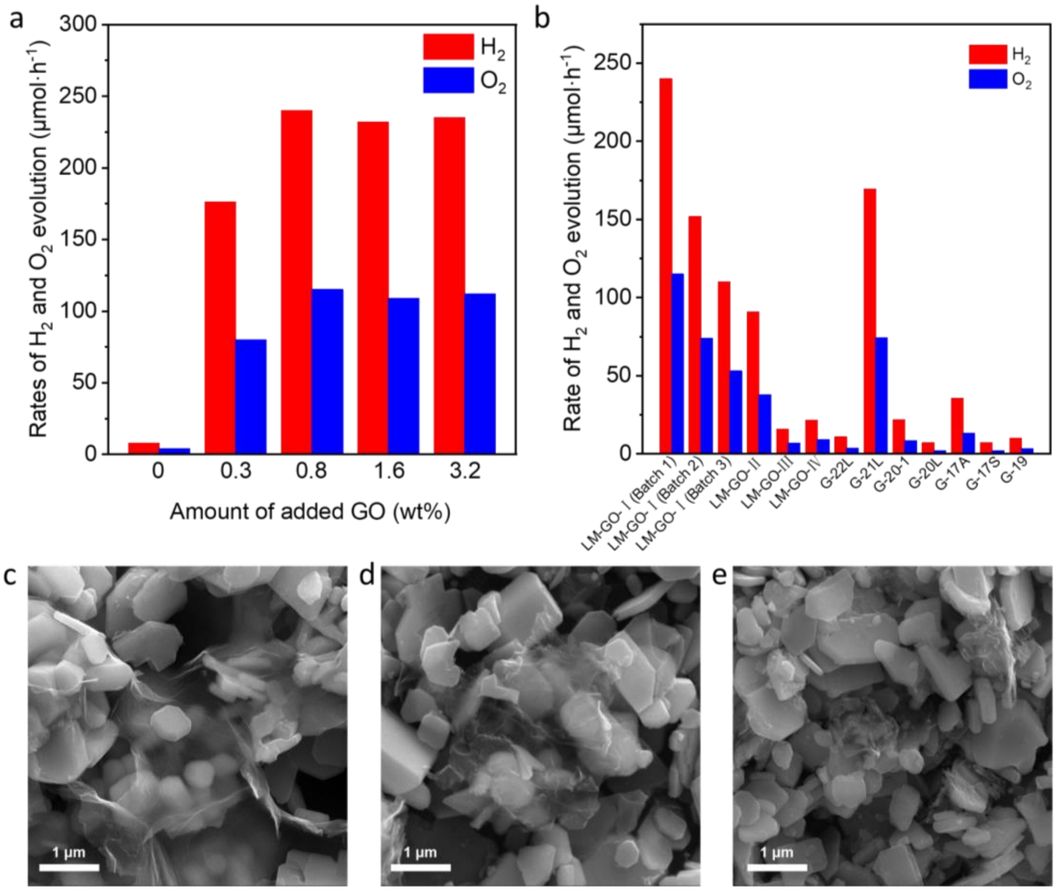

Supplementary Figure 18. Effect of GO on performance. a, OWS activity as a function of the mass of GO. **b,** OWS activities obtained with different types of GO. Typical morphologies of Z-scheme systems incorporating **c,** LM-GO-I (Batch 1), **d,** G-21L and **e,** G-17A.

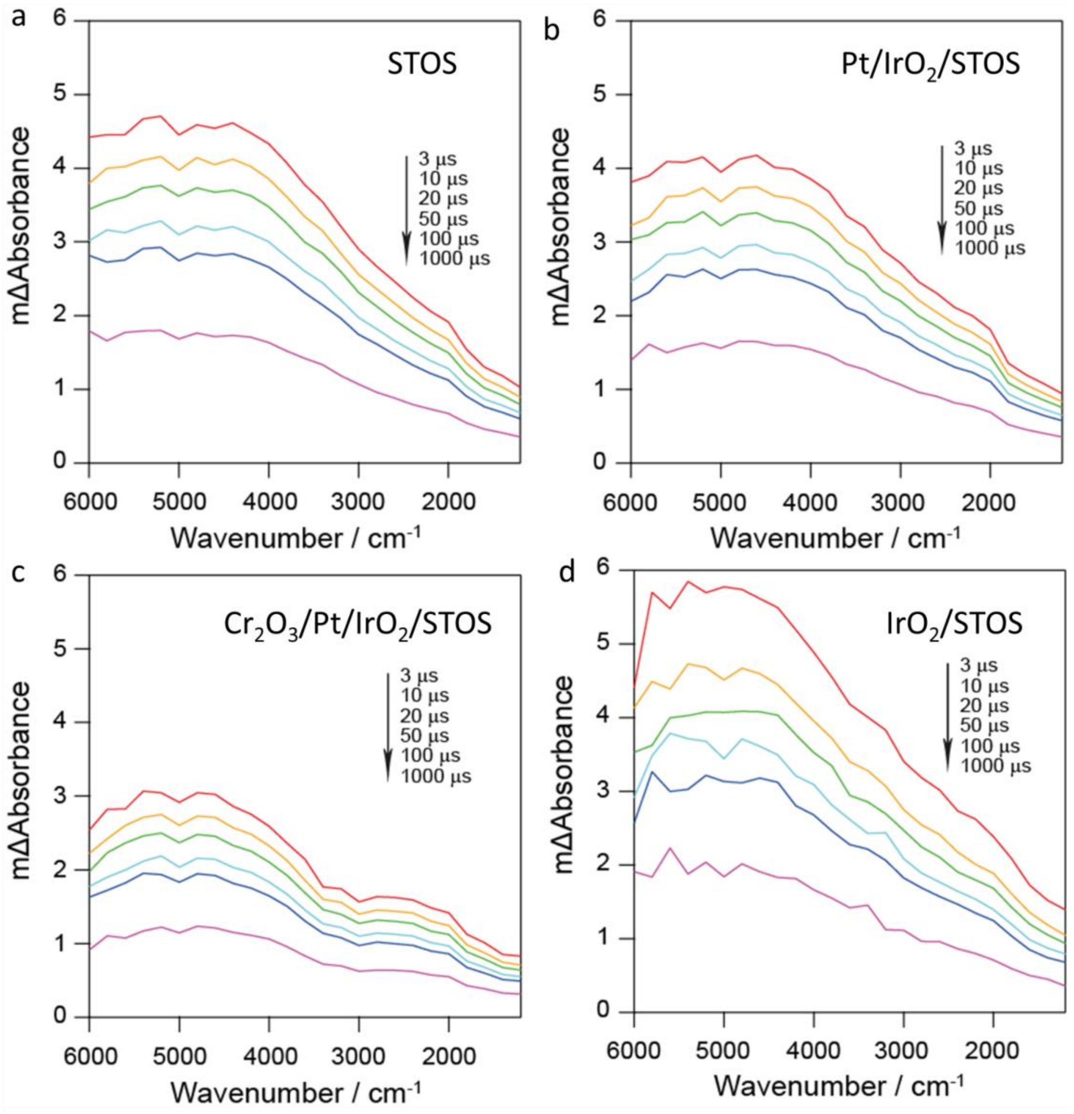

Supplementary Figure 19. TAS analysis of HEP with sequential surface modifications. Mid-infrared transient absorption spectra of photocarriers in the **a,** bare STOS, **b,** Pt/IrO_2/STOS, **c,** Cr_2O_3/Pt/IrO_2/STOS and **d,** IrO_2/STOS. Samples were excited by 470 nm laser pulses (duration: 6 ns, fluence: 1 mJ/pulse, frequency: 1 Hz). Data were acquired under N_2 (20 Torr) and at room temperature.

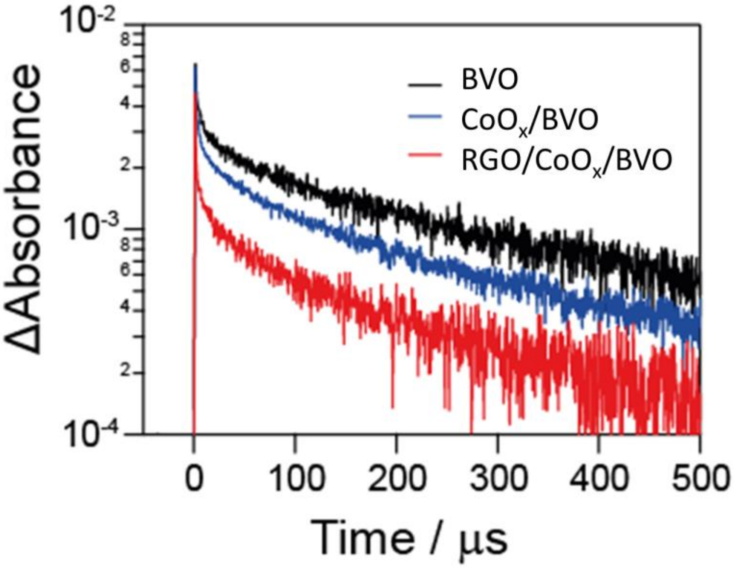

Supplementary Figure 20. Decay kinetics of photocarriers as determined by TAS.

Decay kinetics of BVO, CoO_x/BVO and RGO/CoO_x/BVO probed at 505 nm.

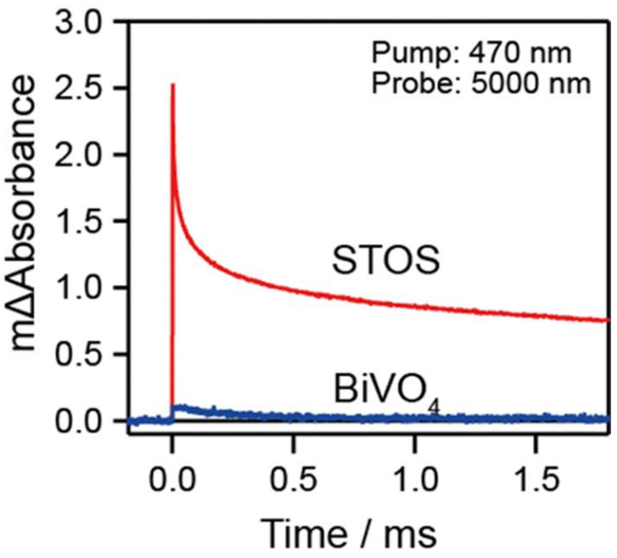

Supplementary Figure 21. TAS characteristics of STOS and BVO samples. Comparison of the TAS signal intensities of STOS and BVO at 5000 nm in response to pump irradiation at 470 nm.

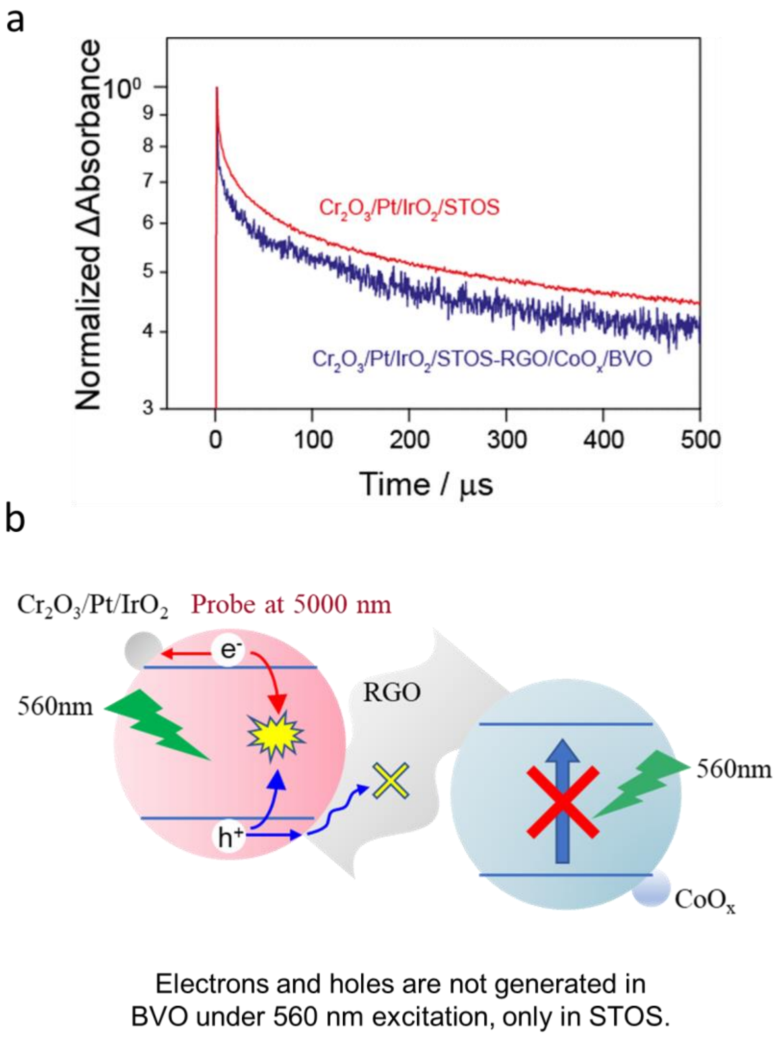

Supplementary Figure 22. TAS analysis of Z-scheme system. a, Decay kinetics of carriers in the Cr_2O_3/Pt/IrO_2/STOS and Cr_2O_3/Pt/IrO_2/STOS-RGO/CoO_x/BVO in response to pump irradiation at 560 nm. **b**, A diagram showing charge transfer between the HEP and OEP in a Z-scheme system.

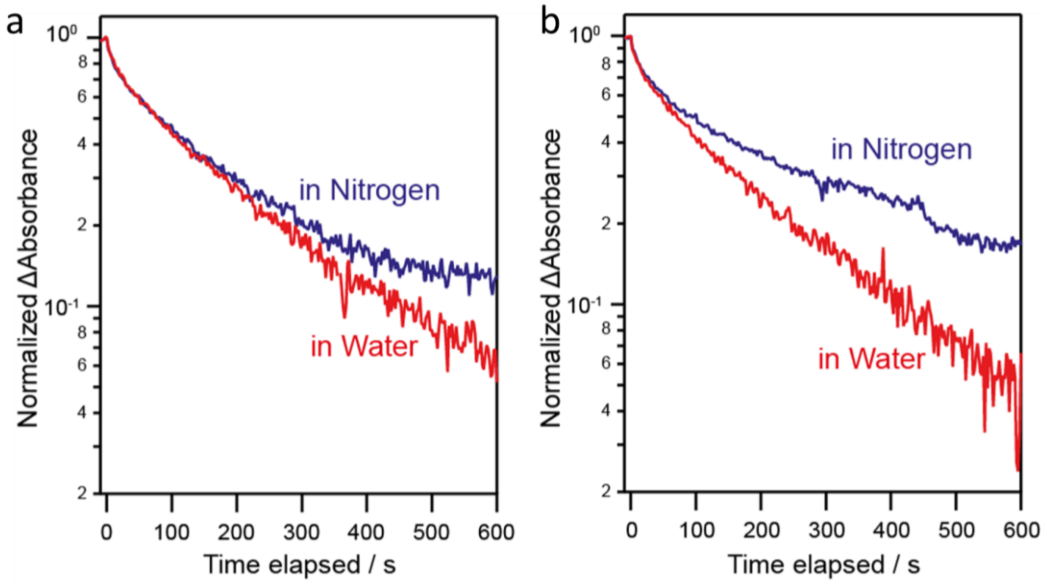

Supplementary Figure 23. Normalized decay kinetics of accumulated electrons in Z-scheme system. a, without and **b,** with RGO. The samples were measured in nitrogen (10 Torr) and water vapor (10 Torr). The samples were excited under continuous irradiation for 223 s using 470 nm CW-LED as the light source and monitored under dark condition (after turning off LED).

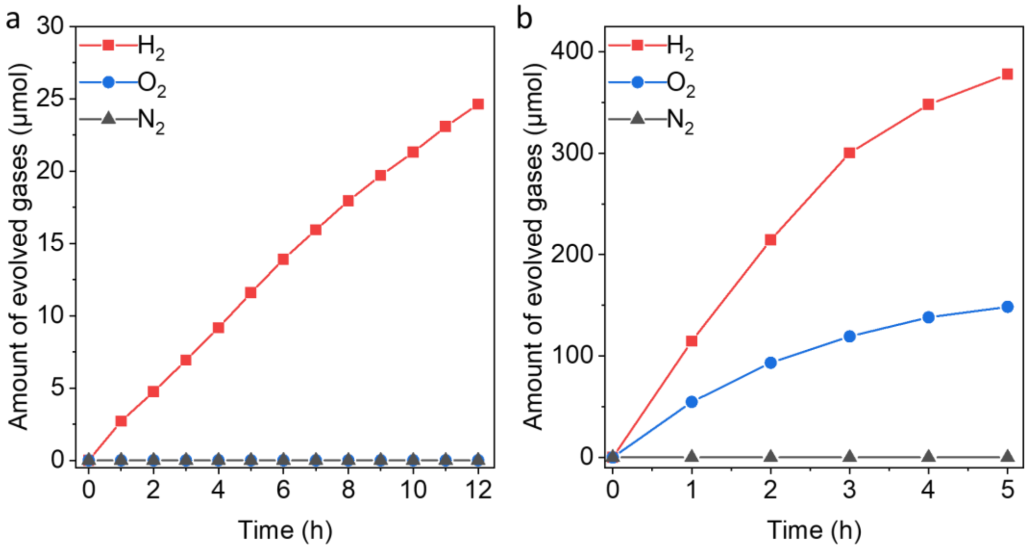

Supplementary Figure 24. Effect of electron mediator on performance. Evolution of gases from a Z-scheme system over time using **a,** I_3^-/I^- and **b,** Fe^{3+}/Fe^+ as the ionic couples.

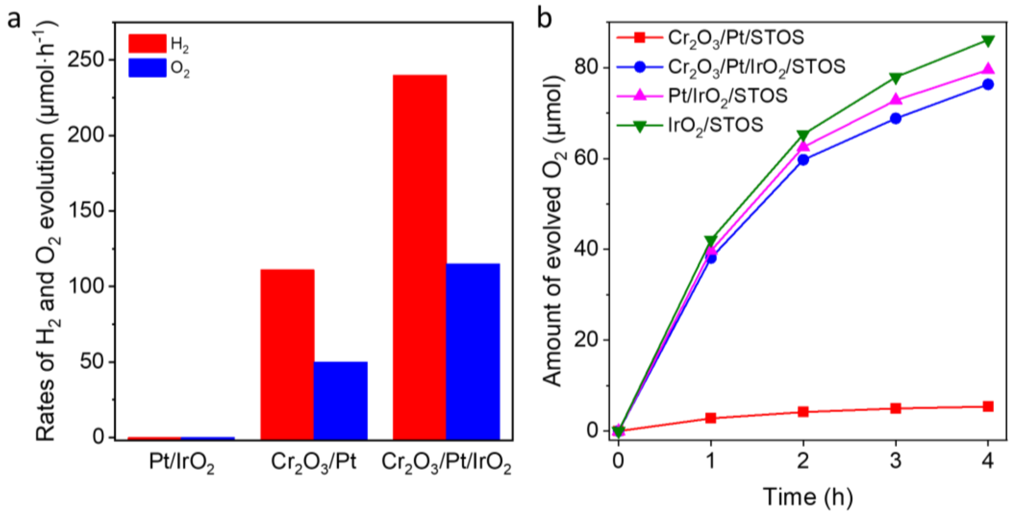

Supplementary Figure 25. Effect of various catalyst modifications on performance.

a, The activities of Z-scheme systems incorporating HEPs modified with Pt/IrO_2, Cr_2O_3/Pt or $Cr_2O_3/Pt/IrO_2$. **b,** Amount of evolved O_2 over STOS modified with IrO_2, Pt/IrO_2, $Cr_2O_3/Pt/IrO_2$ or Cr_2O_3/Pt in O_2 evolution half reaction.

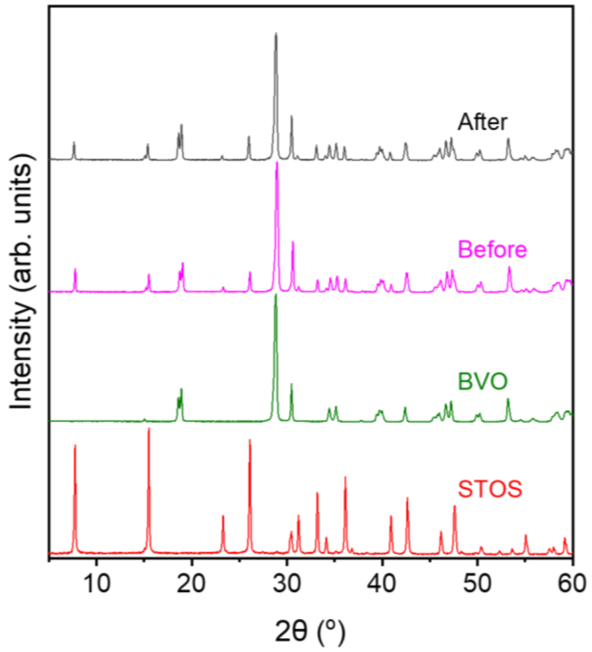

Supplementary Figure 26. Characterization of Z-scheme system before and after reaction. XRD patterns obtained from the Cr_2O_3/Pt/IrO_2/STOS-RGO/CoO_x/BVO before and after a 114 h photoreaction along with STOS and BVO patterns as references.

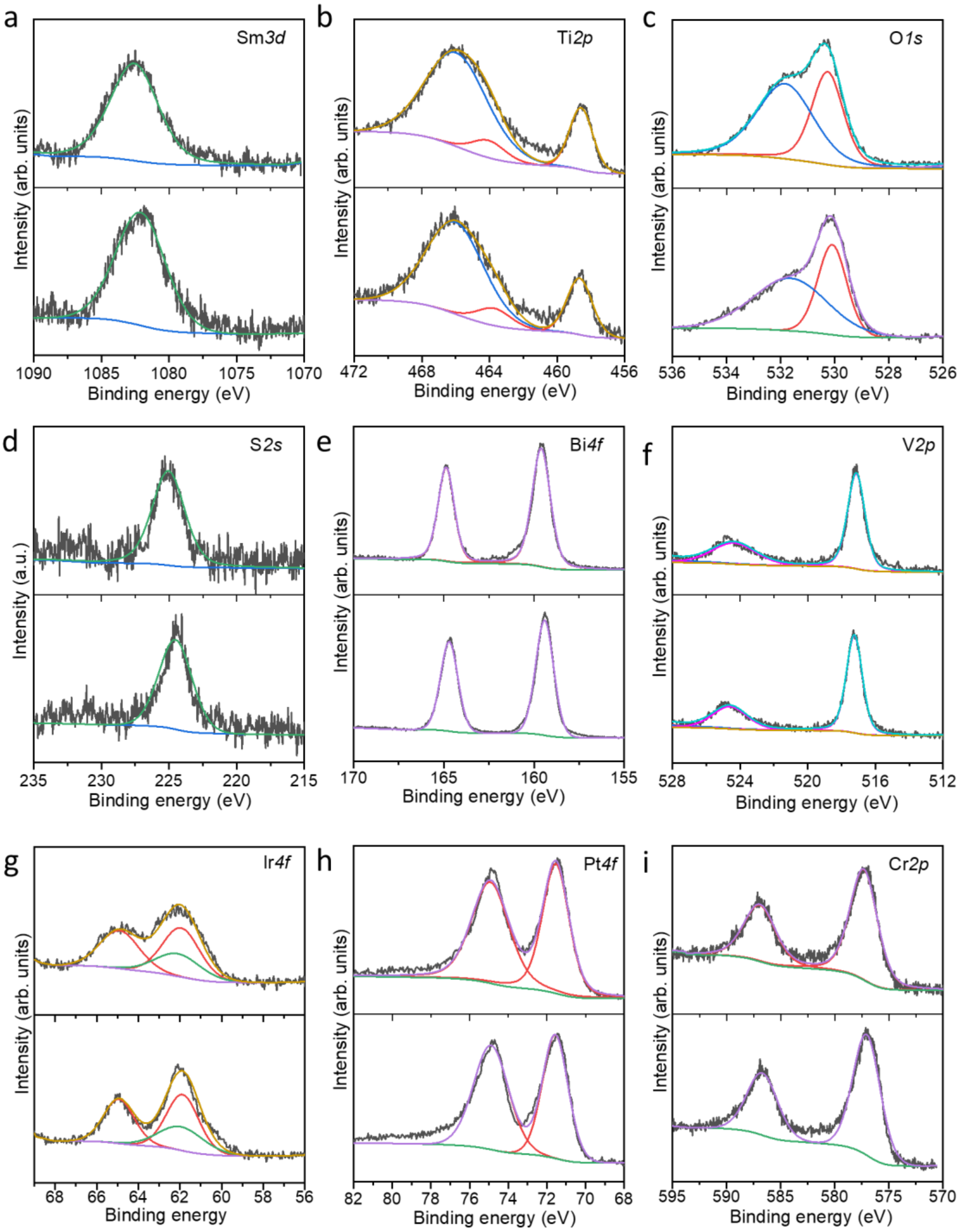

Supplementary Figure 27. Characterization of Z-scheme system before and after reaction. The XPS patterns of **a**, Sm3*d*, **b**, Ti2*p*, **c**, O1*s*, **d**, S2*s*, **e**, Bi4*f*, **f**, V2*p*, **g**, Ir4*f*, **h**, Pt4*f* and **i**, Cr2*p* XPS spectra acquired from the Cr_2O_3/Pt/IrO_2/STOS-RGO/CoO_x/BVO before (upper) and after (lower) a 114 h photoreaction.

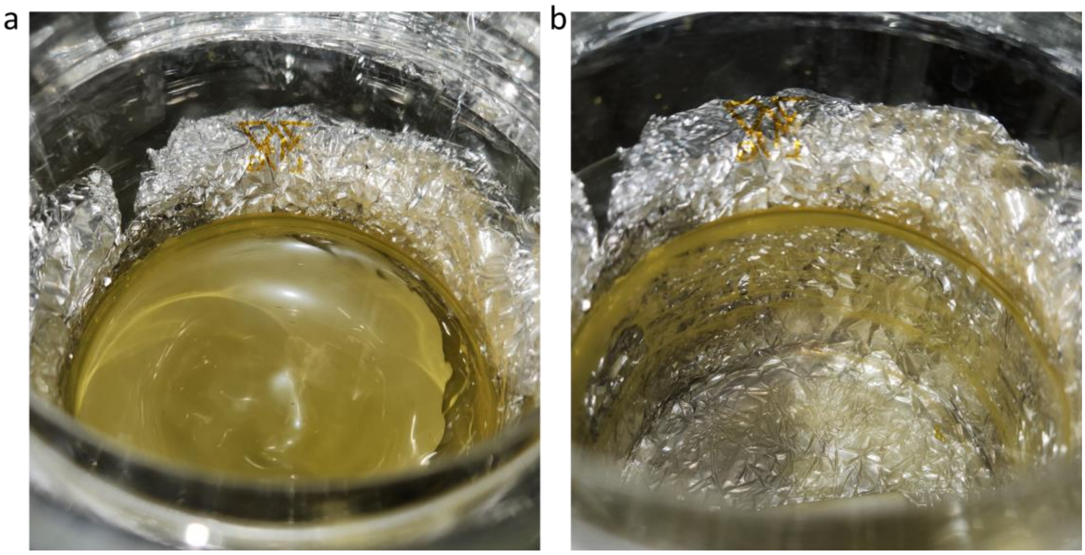

Supplementary Figure 28. Photograph of the reaction after one run of 12 h. The picture of reactor **a.** with aqueous solution and stirring, **b.** after removing the aqueous solution**.**

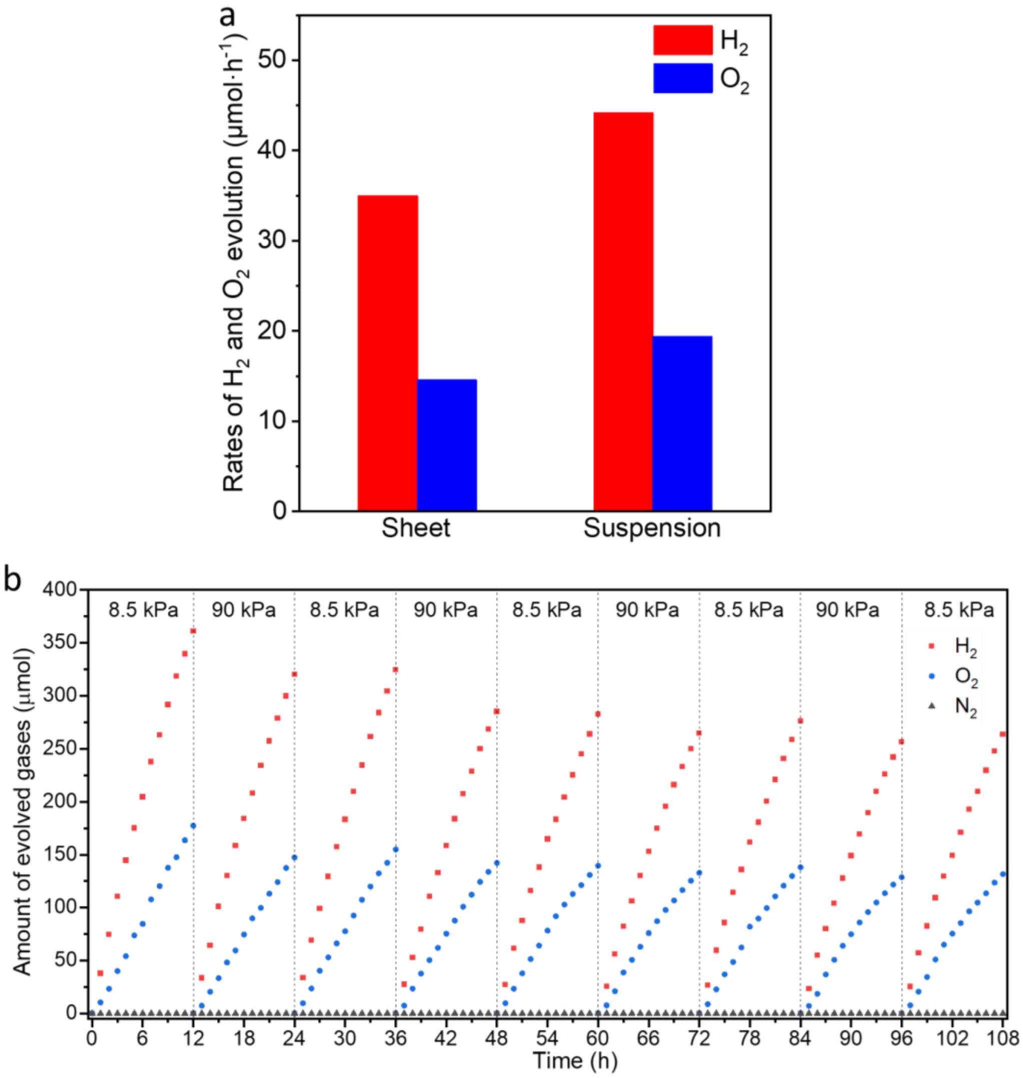

Supplementary Figure 29. Performance of Z-scheme OWS sheet. a. Comparison of sheet and suspension systems under similar reaction conditions (0.02 g photocatalyst, irradiation area of 9.0 cm^2). **b.** Evolution of gaseous products by a Z-scheme system under different Ar background pressures in response to >420 nm light.

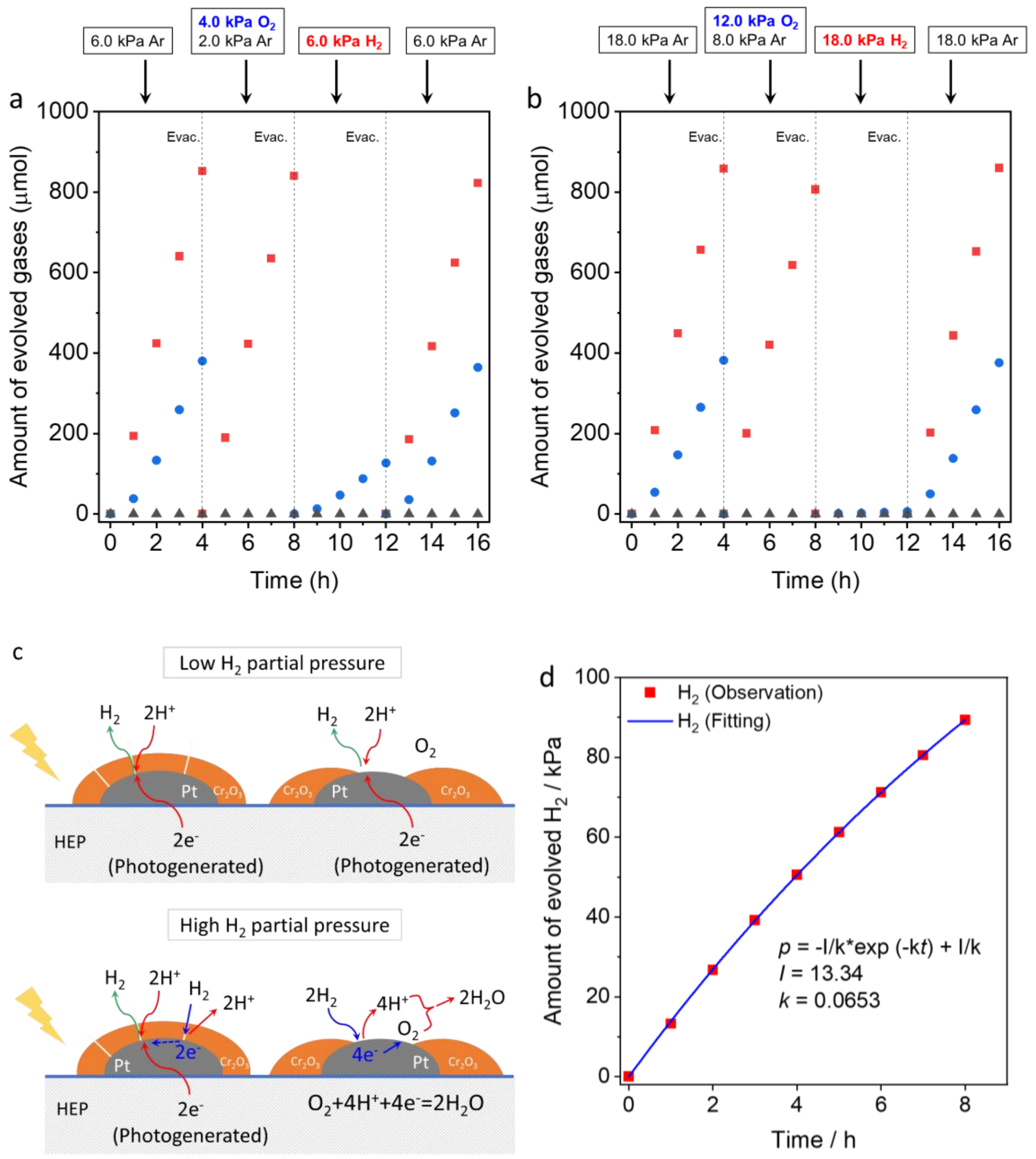

Supplementary Figure 30. Back reaction under light irradiation. a, b. Data used to study the back reaction while introducing varying amounts of H_2 and O_2, respectively. **c,** Proposed mechanism for the back reaction at an increased H_2 partial pressure. **d,** Fitting of a plot of hydrogen evolution from the half-reaction as a function of time assuming a reverse reaction having a first-order dependence on the hydrogen partial pressure.

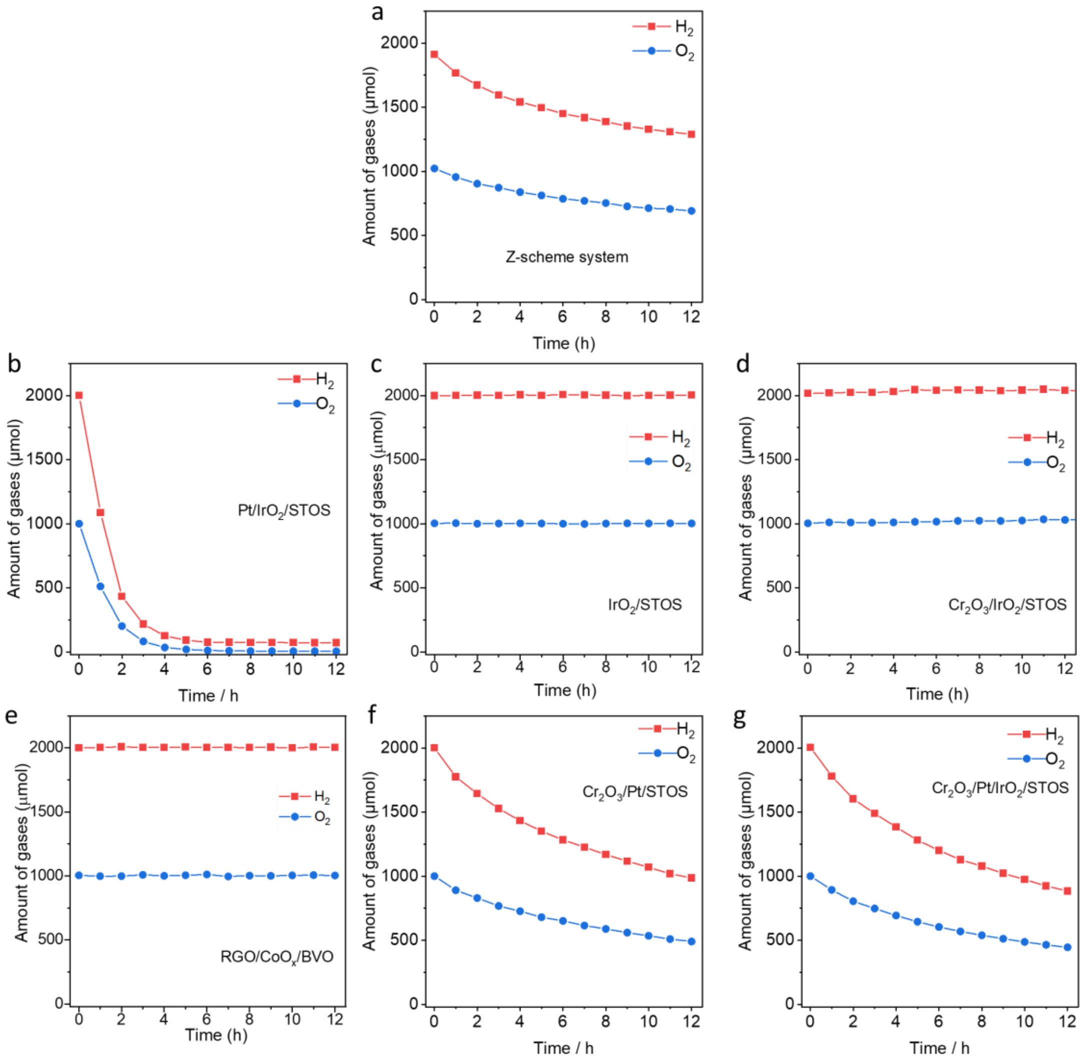

Supplementary Figure 31. Back reaction under dark conditions. Amounts of gases detected in **a,** Z-scheme system and from **b,** Pt/IrO_2/STOS, **c,** IrO_2/STOS, **d,** Cr_2O_3/IrO_2/STOS, **e,** RGO/CoO_x/BVO, **f,** Cr_2O_3/Pt/STOS and **g,** Cr_2O_3/Pt/IrO_2/STOS. Before sampling, around 2000 μmol of H_2 and 1000 μmol O_2 was introduced to the system.

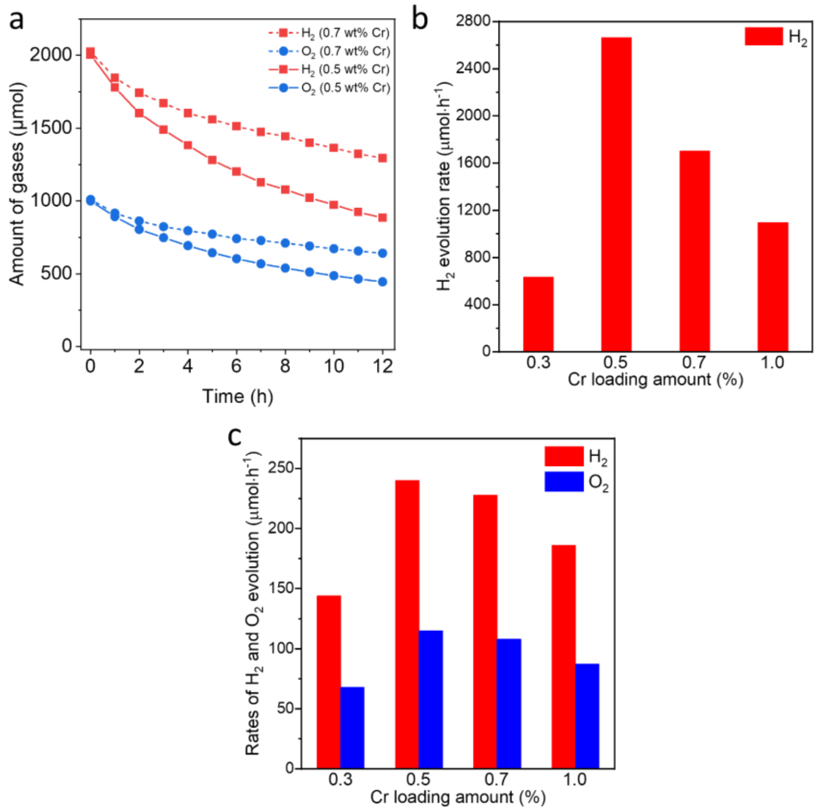

Supplementary Figure 32. Effect of the loading amount of Cr. a, Amounts of gases detected in Pt/IrO_2/STOS with different loading amount of Cr. **b,** H_2 evolution rate of 0.5 wt% IrO_2 and 1.0 wt% Pt modified STOS with different loading amount of Cr and **c,** the corresponding performance in overall water splitting reaction coupled with CoO_x/BVO via RGO.

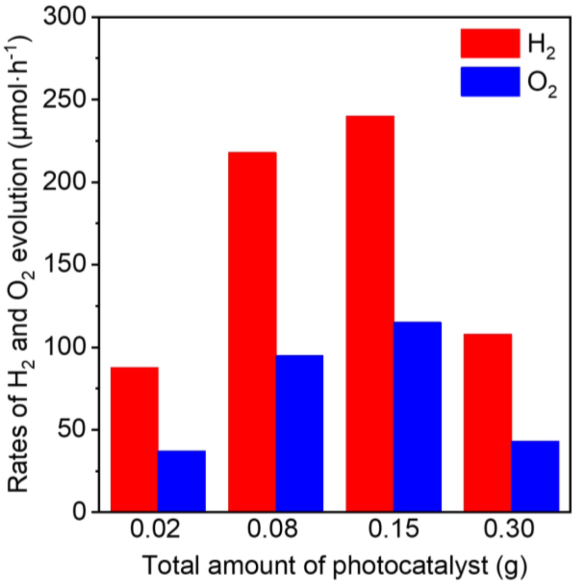

Supplementary Figure 33. Performance of Z-scheme system. Z-scheme OWS activity obtained with varying amounts of Cr_2O_3/Pt/IrO_2/STOS-RGO/CoO_x/BVO. Data were obtained with an HEP:OEP mass ratio of 1:2.

Supplementary Table 1. The amounts of added GO and carbon loaded on the Z-scheme system.

Added amount (wt% vs. BVO)	Detected amount in the Z-scheme system (wt% vs. total mass)	Detected amount in Z-scheme system (wt% vs. BVO)
0.3	0.3	0.4
0.8	0.4	0.6
1.6	0.8	1.1
3.2	1.7	2.5
